# Wear Improvement of Tools in the Cold Forging Process for Long Hex Flange Nuts

**DOI:** 10.3390/ma8105328

**Published:** 2015-09-25

**Authors:** Shao-Yi Hsia, Po-Yueh Shih

**Affiliations:** 1 Department of Mechanical and Automation Engineering, Kao-Yuan University, Kaohsiung 82151, Taiwan; 2 Chong Cheng Fastener Corporation, 69 Da-Shun Rd. Guan-Miau, Tainan 71844, Taiwan; sky999.shih@msa.hinet.net

**Keywords:** cold forging fastener, Archard wear, Taguchi quality method, hex flange nut

## Abstract

Cold forging has played a critical role in fasteners and has been widely used in automotive production, manufacturing, aviation and 3C (Computer, Communication, and Consumer electronics). Despite its extensive use in fastener forming and die design, operator experience and trial and error make it subjective and unreliable owing to the difficulty of controlling the development schedule. This study used finite element analysis to establish and simulate wear in automotive repair fastener manufacturing dies based on actual process conditions. The places on a die that wore most quickly were forecast, with the stress levels obtained being substituted into the Archard equation to calculate die wear. A 19.87% improvement in wear optimization occurred by applying the Taguchi quality method to the new design. Additionally, a comparison of actual manufacturing data to simulations revealed a nut forging size error within 2%, thereby demonstrating the accuracy of this theoretical analysis. Finally, SEM micrographs of the worn surfaces on the upper punch indicate that the primary wear mechanism on the cold forging die for long hex flange nuts was adhesive wear. The results can simplify the development schedule, reduce the number of trials and further enhance production quality and die life.

## 1. Introduction

The widespread use of fasteners reflects their importance in various industries. Taiwan exported roughly 1.46 million tons of fasteners in 2013, with gross revenues reaching U.S. $4 billion to rank fifth in global fastener production. Both the quantity and the quality of fasteners from Taiwan contribute significantly to international markets. However, product innovation is increasingly difficult owing to price competitiveness from China and Southeast Asian countries and to the increasing complexity of emerging products. Fortunately, recent collaborative ventures between academia and industry allow for designers, who require 5–10 years of training, to receive knowledge, expertise and a theoretical foundation through industry-university cooperation. With comprehensive curriculum planning and the introduction and training of operating skills, the practical experiences of designers are combined with a theoretical knowledge of graphics and simulation software, which is then cross-referenced and mutually verified to improve a project. Moreover, peer involvement stimulates manufacturing skills, simplifies the product development schedule, reduces overhead costs and increases industrial competitiveness.

Wear, such as friction loss, erosion and fatigue, commonly damages machine elements. Wear appears between objects when friction causes continuous material peeling and loss. Die wear is of priority concern in manufacturing, because it lowers the quality of work pieces, reduces die life and raises processing costs. Metal forming die wear research largely adopts the Archard wear model [1], which assumes that the wear volume generated over a sliding distance is related to the contact area in order to derive the wear equation and to study the effects of processing parameters on metal fabrication wear. Based on finite element analysis, Vardan *et al.* [2] examined die wear in rough forging and discussed friction mechanisms during metal forming. Wibom *et al.* [3] studied the rising temperature caused by friction and the effects of lubricating conditions on die wear and life. Based on finite element analysis, Lee *et al.* [4] predicted die life during bolt forming and proposed empirical formulas for fatigue failure and wear. According to their results, the fatigue failure mode should be applied to evaluate the die life of high-tensile cold forging steel, while the wear model could be used for evaluating low tensile cold forging steel. Furthermore, several studies on the simulation of cutting die wear discussed wear parameters. Yen *et al.* [5] evaluated wear with the parameters of metal cutting tools (e.g., cutting temperature, contact stress and relative sliding speed), indicating that such parameters play major roles in tool wear. Dubar *et al.* [6], Bouzakis *et al.* [7] and Filice *et al.* [8] also analyzed wear in coated cutting tools. Stavropoulos *et al.* [9] investigated the limitations of tool wear prediction on the milling of CGI 450 plates through the simultaneous detection of acceleration and spindle drive current sensor signals. Their results indicate that predictability is affected by the mean signal energy acquired from vibration acceleration signals.

It has become a common trend in recent years to use computer aided analysis software FEM (DEFORM-3D) for the development of metal forging processes. Its introduction into the design and production of fastener dies could effectively shorten product development time by reducing the number of die tests and test failures. Vazquez *et al.* [10] summarized an investigation of different alternatives for improving the life of a tungsten carbide insert used in a cold forming operation performed on an automatic cold header. Their analysis of metal flow and stress on the carbide insert used FEM software to improve insert life. Vazquez and Altan [11] also combined computer aided engineering with physical modeling for applications with cold forging connecting rods. Modifying the forming process based on DEFORM analysis software results, production costs were effectively reduced, tolerance decreased and die life enhanced. Moreover, flow defects generated during product fabrication were predicted for further improvement. Falk *et al.* [12] applied Finite Element DEFORM software to propose a method correlated with forging volume, which easily and accurately predicted cold forging die life and was verified with experiments. Joun *et al.* [13] presented an application-oriented finite element approach to forging die structural analysis. The die set structural analysis problem was formulated as a contact problem considering both shrink fit and preloaded clamping, solved iteratively by a varying penalty method. Hsia [14] used FEM DERORM-3D software to simulate the 3C micro-pin forward extrusion and forging process. His study simulated the forward micro-extrusion of a workpiece, discussed the differences between the simulated stage process and the experimental process and determined effective stress-strain and material flow properties after extrusion, as well as the upper punch reaction when the material was formed. This was used as the evaluation standard for designing an upper punch and determining the strength of a die structure. Hsia and Chou [15] used computer simulations to analyze the fabrication of hexagonal nuts, and experimental metallography and hardness values were used to understand the cold forging characteristics of hexagonal nuts. Their research provided the information needed to understand forging loads and forming conditions at various stages before fastener formation. Han and Lin [16] utilized finite element (FE) methods to predict die and workpiece wear during cold rotary forging by systematically investigating contact pressure and contact slip distance. Consequently, new insight into the wear that occurs at the surfaces of dies and workpieces was gained based on Archard’s wear law.

Many studies have focused on the wear model used in tool design and computer software utilized in the analysis of metal forming processes. However, since the operative wear regime cannot be accurately predicted in a manufacturing process, few scholars have explored wear mechanisms at the interface between tool and workpiece. This results in operator experience and trial and error being subjective and unreliable in the fastener industry. By using computer-assisted product design software and computer-assisted metal forming software in the analysis of long hex flange nuts, this study investigated the major parameters required for die design and stress distribution in a workpiece. Different specimens were used in experiments to evaluate the flow stress-strain curve and friction coefficient. Moreover, based on DEFORM-3D finite element analysis, the production of long hex flange nuts was simulated to determine metal flow, stress distribution, load conditions and optimal wear conditions in order to select optimal die materials and relevant equipment during the primary stage of product development. Finally, multistage serial forged products were used to verify the accuracy of our numerical analyses, and SEM micrographs of the worn surfaces on the upper punch indicate that the primary wear mechanism on the cold forging die for long hex flange nuts was adhesive wear. Consequently, simulations verified by actual fabrication can simplify the development schedule for the entire process, reduce the number of trials and further enhance production quality and die life.

## 2. Research Methodology and Experimental Structure

### 2.1. Research Methodology

**Figure 1:**
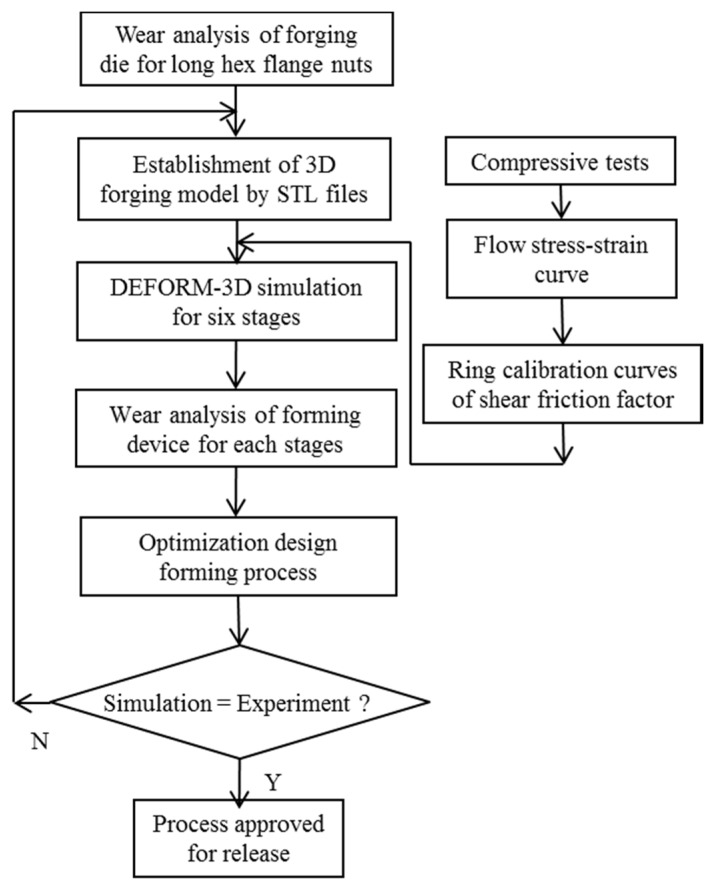
Flowchart of the study.

The mechanical properties of materials were first analyzed. A cylindrical compression test, which included a flow stress test, was then performed to determine the DEFORM-3D pre-processor die setting. Die preform design and relevant research procedures are also discussed. Our research process is illustrated further in a flowchart (Figure 1). When experiments did not conform to simulation results, the flow stress-strain curve, ring calibration curves of the shear friction factor or mesh situation were reassessed.

### 2.2. Mechanical Properties of Metal Materials

In the DEFORM analysis, an accurate flow stress-strain curve is an effective method of evaluating metal forming. It can indicate that the metal starts flowing or deforming plastically when the applied stress (in uniaxial tension without necking and in uniaxial compression without bulging) reaches the value of the yield stress or flow stress. Because strain ranges during forge forming are broad and the pretreatment processes of materials are different, compression tests are required to determine actual flow stress-strain curves. The compression test is performed to calculate the stress-strain curve of actual materials, which enables DEFORM to describe forming characteristics with a constitutive equation to increase simulation accuracy and reduce simulation errors.

The relationships among flow stress, strain, strain rate and temperature can be expressed as:

(1) $$\bar{\sigma }=f\left(\bar{\varepsilon },\;\dot{\bar{\varepsilon }},\;T\right)$$

where $\bar{\sigma }$ denotes flow stress, $\bar{\varepsilon }$ represents strain, $\dot{\bar{\varepsilon }}$ refers to the strain rate and *T* is temperature. Normally, when the metal processing temperature is higher than the recrystallization temperature, strain does not appear to significantly affect flow stress, while the strain rate affects flow stress considerably more. When the processing temperature is lower than the recrystallization temperature, the effects of strain (*i.e*., work hardening) become more important, and the extent to which the strain rate affects flow stress can be neglected. In this study, the cold forging process did not appear to be affected when a punch speed of 5 mm/min was used in the compression test.

The material and characteristics of the die for forming long hex flange nuts (e.g., upper punch, lower punch, die and shell) are described below.

(1) Upper punch, high-speed steel SKH55 is characterized by high wear resistance, sintering resistance and high compressive strength.
(2) Lower punch, SKH9 is characterized by high wear resistance, high compressive strength, excellent surface treatment processing and temper softening resistance.
(3) Die, WC is characterized by extremely high hardness, wear resistance and high compressive strength.

Table 1 lists the characteristics of the above materials.

**Table 1:** Software parameter settings.

	Workpiece	Upper Punch	Die	Lower Punch
Properties	Elastic-plastic	Rigid		
Material	C1010	SKH55	WC	SKH9
Mesh number	35,000	30,000		
Hardness	HRC38	HRC66	HRC77	HRC61

### 2.3. Establishing the Flow Stress-Strain Curve

#### 2.3.1. Cylinder Experiment

**Figure 2:**
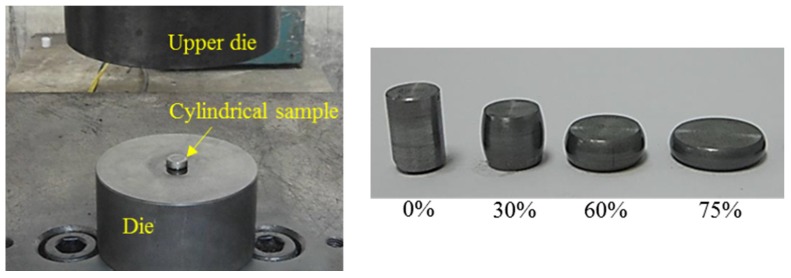
Compression experiment and compressed cylindrical samples at 0%, 30%, 60% and 75% reductions.

Fastener manufacturers acquire workpieces with standard specifications (cylinder diameter 10 mm and height 15 mm) through CNC lathe processing. A cylindrical compression test was then performed with a 100-ton universal hydraulic testing machine in the laboratory (Figure 2). In the cylindrical compression test, the displacement controller of the universal testing machine was first adjusted for compression height. The cold forging process for long hex flange nuts in this study means a fabrication from a bulk material at room temperature with no initial heating of the preform or intermediate stages. After compressing the cylinder to the target height, load and displacement data are read from a computer and substituted in the equation to calculate the true stress-true strain curve.

#### 2.3.2. Calculation of Flow Stress-Strain Curve

**Figure 3:**
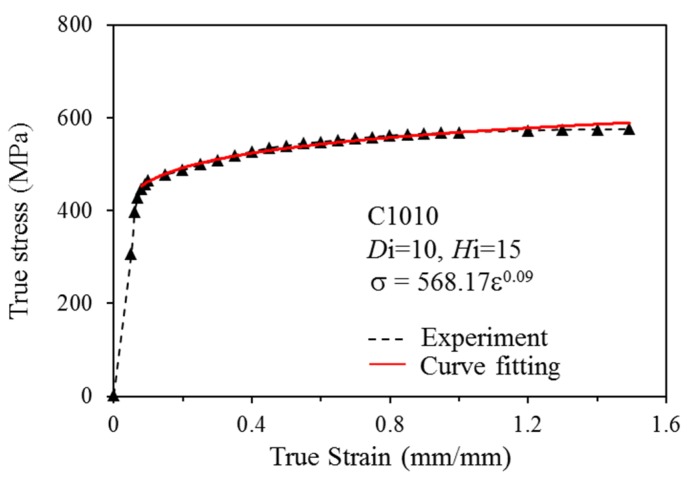
True-stress and true-strain curve of C1010 (*H*_i_ was the initial height of the compressed cylindrical sample, and *D*_i_ was its diameter).

In the cylindrical compression test at room temperature and with lubrication, the received load displacement diagram is used to solve the stress-strain equation for the flow stress curve of the C1010 material under different conditions. The engineering stress-strain equation is:

(2) $$\sigma_{E}=\frac{F}{A_{0}},\;\varepsilon_{E}=\frac{L_{0}-L}{L_{0}}$$

where σ*_E_* is engineering stress, *F* is load, *A*_0_ is cross-sectional area, ε*_E_* is engineering strain, *L* is changed length and *L*_0_ is original length. The true stress-true strain equations are:

(3) $$\sigma_{T}=\frac{F}{A}=\sigma_{E}\left(1-\varepsilon_{E}\right)$$

(4) $$\varepsilon_{T}=-\text{ln}\left(\frac{L}{L_{0}}\right)=-\text{ln}\left(1-\varepsilon_{E}\right)$$

where σ*_T_* is true stress and ε*_T_* is true strain. The flow stress curve of the C1010 material at room temperature can be determined (Figure 3) based on the above equations and the load displacement diagram. When received, the flow stress curve of the material under true forging conditions was imported into the material database in the DEFORM-3D finite element analysis software to more precisely simulate the material’s changes during forging and, thereby, to allow simulation results to better represent realistic conditions. The flow curve derived by statistical regression was represented as σ = 568.17ε^0.09^.

### 2.4. Measurement of the Constant Shear Friction Factor

#### 2.4.1. Establishment of the Constant Shear Friction Factor Calibration Curve

By using DEFORM-3D, material properties were simulated for the ring compression test. Additionally, the constant shear friction factor calibration curve was established based on simulation results under given friction conditions, as well as the ring inside diameter reduction ratio and the height reduction ratio measured after the compression test. When the inside diameter of a circle is determined, the circle of the center can be calculated using the perpendicular bisectors of two chords. Then, two points passing through the circle center are the diameter of the inner circle, where the equation to calculate the inside diameter reduction ratio is shown as follows:

(5) $$\frac{r_{\text{o}}-r}{r}=\text{reduction in internal diameter }(\%)$$

where *r* is the ring radius after compression and *r*_0_ is the original ring radius.

Ring height is measured next. Reduction data were acquired at compression step frequencies of 20, 40, 60, 80 and 100, and corresponding inside diameter reduction ratios were calculated for the calibration curve under constant shear friction factors. However, simulation data can be acquired by setting the simulation temperature below 20 °C. When the constant shear friction factors were 0.12, 0.15, 0.17, 0.20, 0.25 and 0.3, the corresponding inside diameter reduction ratios and height reduction ratios after compression were cross-analyzed with the experimental results to establish the constant shear friction factor calibration curve of the C1010 material.

#### 2.4.2. Ring Compression Test

The fastener company’s standard ring compression test specifications for outside diameter to inside diameter to height were 6:3:2, yet the actual size of the outside diameter was 18 mm, inside diameter 9 mm and height 6 mm. Compression tests with and without lubricating conditions were done at room temperature.

**Figure 4:**
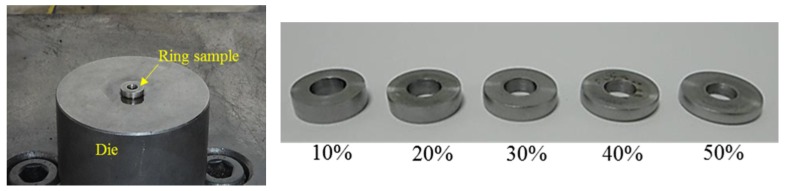
Compressed ring samples at 10%, 20%, 30%, 40% and 50% reductions.

**Figure 5:**
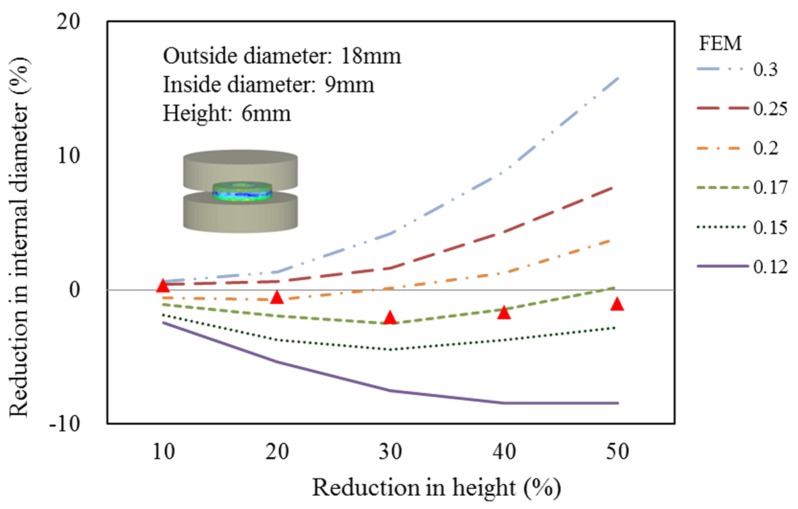
Ring calibration curves obtained with σ = 568.17ε^0.09^ (MPa).

In the ring compression test, the upper round holder block was adjusted to make contact with the ring. The machine was then set at zero to start the ring test by upsetting the billet at the ring height reduction ratios, which were 10%, 20%, 30%, 40% and 50%. The inside diameters of the ring facing 0°, 30°, 60°, 90°, 120° and 150° were measured, revealing that the inside circumference was not a true circle. Therefore, the mean of the six diameter measurements was assumed to be the inside diameter of the deformed ring (Figure 4). Additionally, ring compression testing was based on DEFORM-3D to obtain the friction coefficient calibration curve. Comparing simulation and experimental results revealed the constant shear friction factor calculated from the inside diameter to height reduction ratio. Figure 5 compares the ring compression test and DEFORM-3D simulation results, which indicated that the inside diameter of the C1010 material was reduced inwards under a high constant shear friction factor. In contrast, the inside diameter expanded outward under a low constant shear friction factor. Approximately 0.17 was assumed to be the base from which the inside diameter expands outward when the constant shear friction factor <0.17; meanwhile, it contracted inward when the constant shear friction factor was >0.17. Therefore, a constant shear friction factor of 0.17 was used to achieve better simulation results.

### 2.5. DEFORM Simulation Parameter Planning

The DEFORM pretreatment setting merely considered forming and not thermal conduction. In the forming process, all die parts, except the workpiece, were set as rigid bodies at a cold forging temperature of 20 °C and the C1010 material was used. Our experimental results established the flow curve equation of the actual workpiece to be σ = 568.17ε^0.09^. Punch speed was set to 253 mm/s downward and each step set to 0.1 mm. The friction coefficient was set to 0.17 with a constant shear friction mode according to the experimental results. The wear analysis in DEFORM was performed using the Archard wear theory, which is the only one that is broadly accepted and used in metal forming. Additionally, the wear equation was a simple model used for describing sliding wear and based on asperity contact theory. The Archard wear theory calculation equation is:

(6) $$V=k\frac{L\times P}{H}$$

where *V* is the wear volume, *k* is the wear coefficient, *L* is the sliding length, *P* is normal pressure and *H* is hardness (HRC used in this study). The wear coefficient may vary several orders of magnitude due to changes in the predominant wear mechanisms (or wear regimes) caused by slight variations of wear parameters, environment, temperature and other factors. Since the operative wear regime cannot be accurately predicted for the cold forging process, a wear coefficient of 1 × 10^−6^ was used in this simulation [17,18]. From the equation:

(1) The wear volume of the material is proportional to the sliding distance,
(2) The wear volume of the material is proportional to the load,
(3) The wear volume of the material is inversely proportional to hardness and,
(4) The wear volume of the material is irrespective of sliding speed.

Fabrication must be done at room temperature in the cold forging process. The rising temperature of the workpiece caused by plastic deformation is minute to the extent that the effects of local temperature change on the forming process are ignored in the forming analysis and the die stress analysis. However, only the thermal conduction mode is turned on in the forming die wear analysis. The workpiece is set as an elastoplastic body, and the rest is set as rigid bodies.

### 2.6. Preform Design and Planning

**Figure 6:**
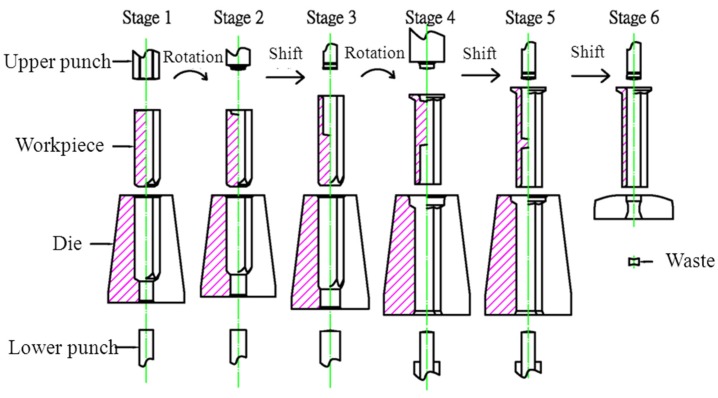
Schematic diagram of six-stage cold forging.

This product was originally an automotive repair fastener (Figure 6). Based on the design of a six-stage nut fabrication machine whose open cutting die is likely to result in uneven notches, a plane punch was used to produce a one-piece material at the first stage. The die bed was then used to remove deformed and sharp edges. During the first stage upsetting, the acute angle of the bed edge covered the center of the workpiece, which was turned over at the second stage. An upper punch created the punch lead hole at the second stage to ensure punch drilling concentricity at the third stage, and the workpiece was then transported to the third stage. Backward extrusion at the third stage at an aperture of ϕ6.9 mm extended the nut to a stroke of 19.5 mm, and the stress caused by backward extrusion was used to form the volume required for the flange surface on the die bed, which was then turned over and moved to the fourth stage. The punch at the fourth stage was used to upset the flange, in which the required geometric size was given for the punch lead hole at the next stage in order to ensure that the punch drilled at the fifth stage was similar to drilling concentricity at the third stage and to prevent piercing at the sixth stage causing burrs by not being concentric. The upsetting volume at the fourth stage was calculated in order to determine the optimal height and thickness (≤1.5×) of the bed design at the third stage, thereby preventing the upsetting from bucking. As the workpiece inner hole was formed at the third stage, the forging load in the flange upsetting was calculated so as to not generate as large a forging stress as an ordinarily closed forging in order to increase stamping die life. The backward extrusion aperture at the fifth stage was ϕ6.9 mm, which extended the nut in a long stroke of 19.85 mm. Inner-hole fabrication further achieved the length and size that conformed to specifications. External die piercing occurred in the sixth step.

### 2.7. Taguchi Quality Method

In the Taguchi experimental design, quantified experimental results are called quality characteristics and can achieve ideals by determining the controlling factors in the experimental design. In order to solve a problem, an engineer needs to fully understand the characteristics of product quality and problems and organize the levels of quality with a fishbone diagram or equivalent. To conduct the experiment at the least cost, an orthogonal array should be selected based on control factors and the levels needed to achieve the required quality with the most precision. The *S*/*N* ratio is then used as the quality index in the Taguchi method to demonstrate the effects of any errors at the process or product level. According to different quality characteristics, various *S*/*N* ratio formulas are formed, including nominal-the-best, smaller-the-better and larger-the-better. The smaller-the-better was applied in this study for the optimization simulation to search for minimum wear, the *S*/*N* ratio [14,15] being:

(7) $$S/N=-10\;\text{log}\left({\bar{\text{y}}}^{2}+{\text{S}}_{\text{n}}^{2}\right)$$

where *y_i_* is the measured value and *n* the number of repeated measurements.

The response table and response graph of each factor were constructed after the experiment to understand the effects on the target function. Based on the *S*/*N* ratio of each factor at the same level of mean calculations, the effects of the factor level on the results were listed in a table and transformed into an auxiliary response graph following:

(8) $$M_{ij}=\frac{\sum_{k=1}^{N}{\frac{S}{N}}_{ijk}}{N}$$

*M*_ij_ is the mean of the *S*/*N* ratio containing *I* factors and *j* levels, *k* is the *k*-th *S*/*N* ratio with *i* factors and *j* levels and *N* is the number of experiments with *i* factors and *j* levels.

After completing the response table, the importance of the control factors at each level of quality was evaluated by:

(9) $$\text{η}\left(Ai,\;Bj,\;Ck,\;Dl\ldots ..\right)={\bar{\eta }}_{Ai}+{\bar{\eta }}_{Bj}+{\bar{\eta }}_{Ck}+{\bar{\eta }}_{Dl}+\ldots -\left(n-1\right)\bar{\eta }$$

where η is the quality objective and ${\bar{\eta }}_{Ai}$ and $\bar{\eta }$ the response value and mean, respectively, in the response table. Equation (9) was used to predict the results of all combinations of inputs tested.

**Figure 7:**
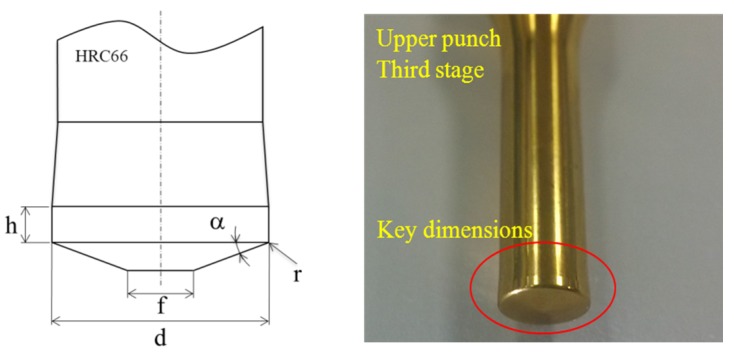
Key dimensions of the third stage upper punch for optimization design.

According to a fastener corporation, the upper punch at the third or fifth stage always had the most wear during the cold forging of long hex flange nuts. Hence, this study focused on optimizing those two stages. By following the Taguchi method (Table 2), flat top (*f*), ring height (*h*), oblique angle (α), arc length (r) and outer diameter (d) were used as key dimensions for designing the upper punch (Figure 7; in this table, italics refer to the original design being: 0.0 mm top flat, 0.7 mm head height, 8° oblique angle, 0.3 mm arc length and 6.9 mm outer diameter). All five factors have four levels each that were used to determine the effects on cold forging extrusion under varying conditions and to optimize the design. Sixteen sets of Taguchi’s orthogonal array *L*_16_(4^5^) were applied to acquire the forming processes. The 16 sets contain five factors, each of which has four levels for an experiment.

**Table 2:** Five factors and three levels of the orthogonal array *L*_16_(3^5^).

Factors	Specifics	Level 1	Level 2	Level 3	Level 4
A	*f* (ϕ, mm)	0	1.725	3.45	5.175
B	*h* (mm)	0.5	*0.7*	0.8	1
C	α (Deg)	*8*	10	12	15
D	*r* (mm)	0.3	0.5	0.7	1
E	*d* (ϕ, mm)	6.71	6.76	6.82	*6.9*

Values in italics represent the original design.

## 3. Results and Discussion

### 3.1. Load Analysis of the Preform Design

**Figure 8:**
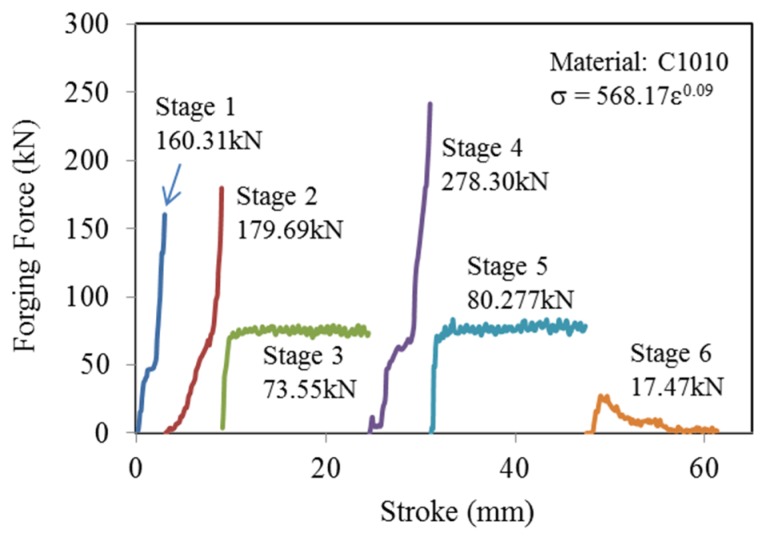
Forging loads of the six stages.

This section analyzes the die fabrication of long hex flange nuts and simulates forging loads from the first to the sixth stages (Figure 8). Forging loads at various stages are shown below. The die approached closed forging during workpiece first-stage reshaping, during which the forging load (160.31 kN; 20.3%) was obviously larger than other loads. The engineering resembled the first stage during reshaping and forming the lead hole at the second stage, the forging load being 179.69 kN (22.77%). In the long stroke (19.5 mm) backward extrusion at the third stage, the forging load (73.55 kN; 9.31%) appeared to linearly increase because the material flow space was not restricted, and the load proportion was <10%. The forging load (278.30 kN; 35.24%) was largest among all stages during flange forming with a closed upsetting at the fourth stage. With the same engineering as the third stage, the extending stroke at the fifth stage was 19.85 mm and the forging load 80.28 kN (10.16%), also revealing linear growth. The forging load (17.47 kN; 2.21%) in the simple via-hole at the sixth stage was less than in the other stages. Overall, the total forging load for long hex flange nuts from the first stage to the sixth stage was 789.78 kN.

### 3.2. Preform Design Wear Analysis

This section analyzes the preform design to determine wear during the forming of long hex flange nuts. The positions of maximal die wear depth at each stage were assessed according to workpiece flow (velocities, strain rates and strains) and stress concentration, the results being used to predict fabrication die life.

**Figure 9:**
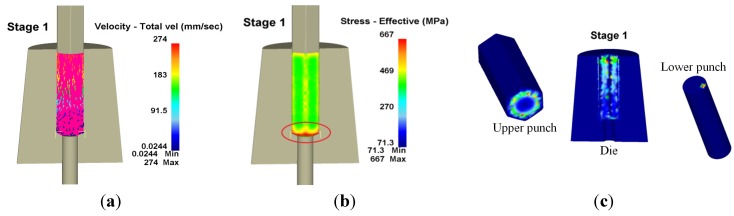
Velocity, effective stress and wear distribution of the first stage in 3D. (**a**) Velocity; (**b**) Effective stress; (**c**) Wear.

Regarding workpiece reshaping at the first and second stages, the punch upsetting determined workpiece flow in the die and flow direction changes at turning corners owing to the geometric constraints of the die. Therefore, stress was concentrated in this area, and there appeared to be more wear where the die contacted the workpiece. Velocity, effective stress and wear distribution of workpiece flow within the forming die in the first stage are shown in Figure 9, where the red circle denotes the area with the largest wear. The largest wear depth appeared on the inner angle of the die chamfer, with a wear depth of 48.6 nm, a maximum wear depth at an upper punch of 7.88 nm and a wear depth at a lower punch of 0.76 nm. According to Equation (6), the die wear is affected by the sliding length and normal stress in the forming simulation.

**Figure 10:**
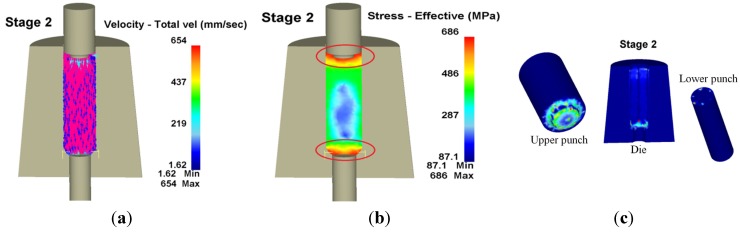
Velocity, effective stress and wear distribution of the second stage in 3D. (**a**) Velocity; (**b**) Effective stress; (**c**) Wear.

Figure 10 shows the workpiece velocity, effective stress and wear distribution in the second stage, where the red circle refers to the areas that had the largest wear. The greatest wear depth occurred on the inner angle of the die chamfer, with a wear depth of 71.4 nm, a maximum wear depth at the upper punch of 109 nm and a maximum wear depth at the lower punch of 3.83 nm.

**Figure 11:**
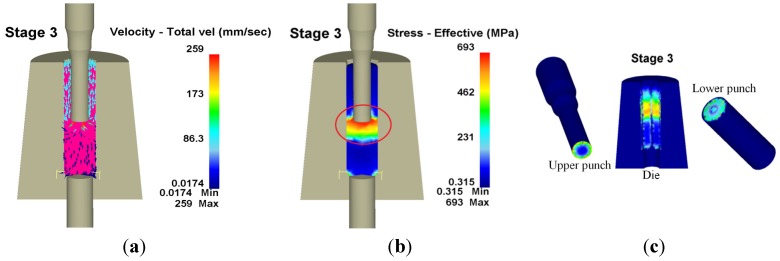
Velocity, effective stress and wear distribution of the third stage in 3D. (**a**) Velocity; (**b**) Effective stress; (**c**) Wear.

The punch extrusion direction was opposite the workpiece flow direction and restricted to the die in the third stage. The fillet of the upper punch changed the workpiece flow direction, causing stress to concentrate on the area and inducing the larger wear. Additionally, wear depth increased with extending length, being 19.5 mm during the long stroke at the third stage, but not much different at 19.85 mm measured in the backward extrusion stroke at the fifth stage. Figure 11 shows the workpiece velocity and effective stress, as well as the wear distribution in the third stage, where the red circle represents the area with the largest wear. The largest wear depth appeared on the upper punch at 473 nm, with the maximum die wear depth being 17.9 nm and the maximum wear depth at the lower punch being 47.3 nm.

**Figure 12:**
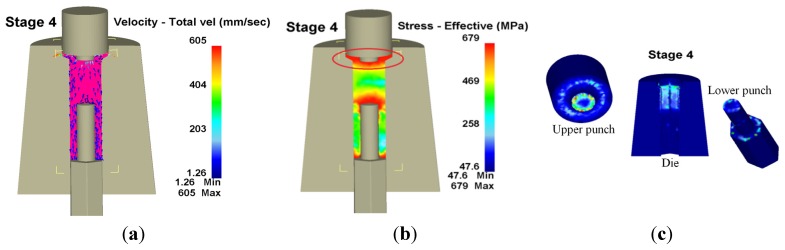
Velocity, effective stress and wear distribution of the fourth stage in 3D. (**a**) Velocity; (**b**) Effective stress; (**c**) Wear.

Figure 12 shows workpiece velocity and effective stress, as well as the wear distribution in the fourth stage, where the largest wear appears in the red circle. Workpiece flow direction was restricted owing to the geometric size of the die during head shape forming in the fourth stage. Additionally, maximal wear depth occurred on the upper punch with a wear depth of 171 nm, a maximum die wear depth at the hex flange of 21.2 nm and a lower punch wear depth of 50.5 nm at its head.

**Figure 13:**
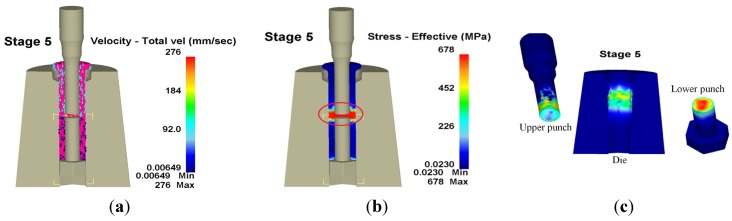
Velocity, effective stress and wear distribution of the fifth stage in 3D. (**a**) Velocity; (**b**) Effective stress; (**c**) Wear.

Figure 13 displays workpiece velocity and stress, as well as wear distribution at the fifth stage, where the largest wear occurs in the red circle. Maximal die wear depth occurred at the upper punch with a wear depth of 472 nm, a maximum die wear depth of 15.4 nm and a maximum wear depth at the lower punch head of 67.3 nm.

**Figure 14:**
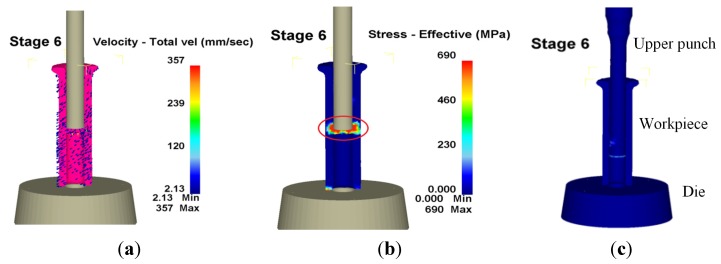
Velocity, effective stress and wear distribution of the sixth stage in 3D. (**a**) Velocity; (**b**) Effective stress; (**c**) Wear.

**Figure 15:**
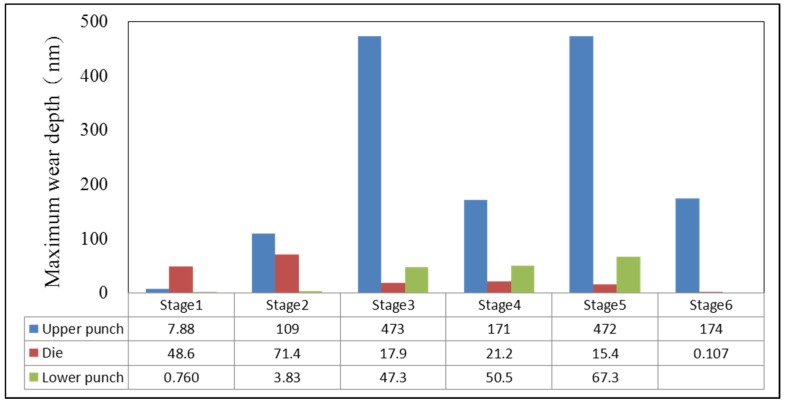
Simulated wear at each stage.

Regarding the via-hole at the sixth stage, the workpiece bed stood up to the die. Furthermore, waste was punched out after the punch top came into contact with the workpiece in which the punch size at the sixth stage became the final aperture of the formed product. Figure 14 presents workpiece velocity, effective stress and wear distribution during the sixth stage, in which the largest wear appears in the red circle. In the via-hole process, friction on the upper punch resulted in it having the maximum wear, with a wear depth of 174 nm. Figure 15 shows the wear of the formed die at different stages; the wear speed of the upper punch is faster than that of the die and the lower punch. Additionally, the most serious wear appeared during backward extrusion in the third and fifth stages. Hence, the next section discusses the optimization of the upper punch during the third stage to reduce the worst wear. Improved performance based on the Taguchi quality method was also achieved in the fifth stage.

### 3.3. Simulating Wear Optimization

**Figure 16:**
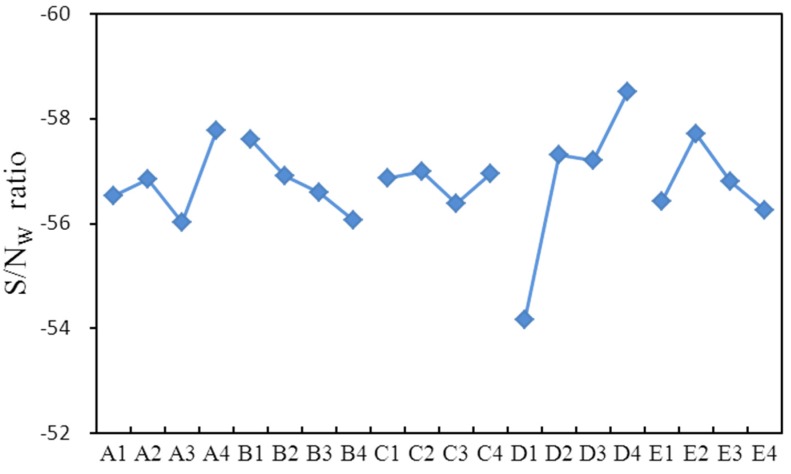
Response graph of *S*/*N*_W_ ratios over five factors (A–D) and four levels (1–4).

Table 3 represents the DEFORM-3D simulations of wear and forging loads and smaller-the-better *S*/*N* ratios according to Taguchi’s orthogonal array *L*_16_(4^5^). The forging load considered was chosen as a reference for comparison with the minimum wear. Table 4 and Table 5 show the factor response tables of the *S*/*N*_W_ ratio on wear and the *S*/*N*_L_ ratio on forging load, which were calculated from Table 3’s simulation results. Level values listed under columns present the effects of variability, and the variability among different levels could be regarded as an effect of controlling factors on *S*/*N*_W_ or *S*/*N*_L_. The range column in Table 4 shows the maximum range of variability, where a larger variability in rank is more important to design optimization. Hence, arc length (*r*) was the largest factor in the simulation results, followed by top flat (*f*), ring height (*h*), outer diameter (*d*) and oblique angle (α) (Table 4). As small *S*/*N*_W_ ratios were considered better (smaller-the-better), the optimal simulation settings for minimum wear were A3B4C3D1E4, implying a 3.45 mm top flat, 1 mm head height, 12° oblique angle, 0.3 mm arc length and 6.9 mm outer diameter. The quality characteristic values in Figure 16 represent the response graph according to Table 4, where x-axis A1 represented the reaction with Controlling Factor A and Level Number 1. The y-axis represented the *S*/*N* ratio of wear. The notation in this figure is similar to that of Table 4. D, A, B and E were significant factors, and C had little influence in all simulations.

**Table 3:** *S*/*N* ratios and minimum loads of the third upper punch using the Taguchi method.

Exp.	Wear (nm)	*S*/*N*_W_ (dB)	Load (kN)	*S*/*N*_L_ (dB)
1	525	−54.40	83.0	−38.38
2	819	−58.27	74.7	−37.47
3	655	−56.32	75.4	−37.55
4	717	−57.11	72.1	−37.16
5	768	−57.71	76.0	−37.62
6	866	−58.75	70.2	−36.93
7	568	−55.09	75.3	−37.54
8	618	−55.82	84.3	−38.52
9	897	−59.06	73.5	−37.33
10	654	−56.31	86.8	−38.77
11	621	−55.86	76.0	−37.62
12	441	−52.89	78.0	−37.84
13	919	−59.27	77.4	−37.77
14	519	−54.30	77.9	−37.83
15	903	−59.11	92.5	−39.32
16	837	−58.45	72.3	−37.18

**Table 4:** Wear response of the third upper punch based on *S*/*N*_W_ ratios.

	A (f)	B (h)	C (α)	D (r)	E (d)
Level 1	−56.53	−57.61	−56.87	**−54.17**	−56.41
Level 2	−56.84	−56.91	−56.99	−57.30	−57.72
Level 3	**−56.03**	−56.60	**−56.38**	−57.20	−56.81
Level 4	−57.78	**−56.07**	−56.94	−58.51	**−56.25**
Range	1.75	1.54	0.62	4.34	1.47
Rank	2	3	5	1	4

**Table 5:** Forging load response of the third upper punch based on *S*/*N*_L_ ratios.

	A (f)	B (h)	C (α)	D (r)	E (d)
Level 1	**−37.64**	−37.77	**−37.53**	−37.90	−38.75
Level 2	−37.65	−37.75	−38.06	−37.84	**−37.38**
Level 3	−37.89	−38.01	−37.81	−37.78	−37.52
Level 4	−38.03	**−37.67**	−37.81	**−37.68**	−37.56
Range	0.39	0.33	0.54	0.21	1.37
Rank	3	4	2	5	1

Table 6 notes the 19.87% improvement in wear that can be applied to the new design in order to maximize die life. The predicted value from additive Equation (9) was also in agreement with the simulation. The new design may induce a larger forging load (4.01%; Table 5). However, the forging load of the third stage was only 9.31% (Figure 8). The increased 4.01% load hence did not greatly affect the cold forging process of long hex flange nuts. Based on experience with the new design from the Chong Cheng Fastener Corporation, a 3.45-mm top flat could present a more uniform punch load and stable metal flow. At the same time, we concluded that arc length played an important role in the long hex flange nut forging die. A smaller arc length of 0.3 mm would induce rapid deformation of the workpiece, whereas an appropriate size can also provide sufficient strength for the cold forming process.

**Table 6:** Comparison of predicted and simulation results between original and optimal designs.

	Wear (nm)		Load (kN)	
	Simulation	Predicted	Simulation	Predicted
Original design	473.00	475.14	73.55	72.14
Optimal design	378.58	384.99	76.50	76.01
Improvement	94.42 (19.87%)	90.15 (18.97%)	−2.95 (−4.01%)	−3.87 (−5.37%)

**Figure 17:**
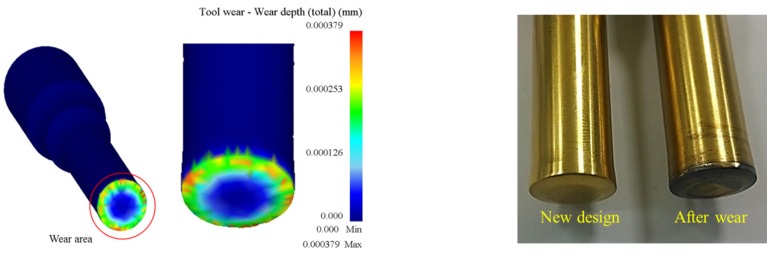
Wear of optimized simulated and actual upper punches.

**Figure 18:**
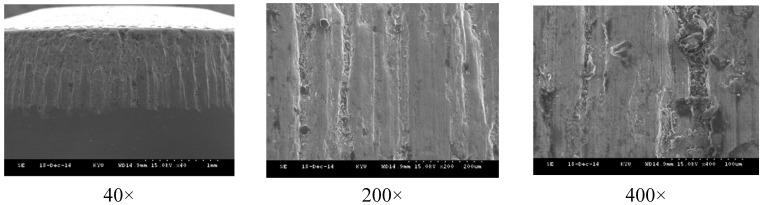
SEM micrographs of the worn surface of an upper punch at various magnifications.

Figure 17 compares the results of simulated and actual wear on the newly-designed upper punch at the third stage where the most serious wear appears. In metal forming, there are four primary mechanisms by which wear may occur; *i.e.*, adhesive wear, abrasive wear, fatigue wear and corrosive/chemical wear. These mechanisms work simultaneously and may be difficult to distinguish during typical metal forming operations. However, Figure 18, which displays SEM micrographs of the worn surface on the upper punch, indicates that adhesive wear may be the primary wear mechanism on the cold forging die for long hex flange nuts.

### 3.4. Complete Product and Simulation Size Measurement

**Figure 19:**
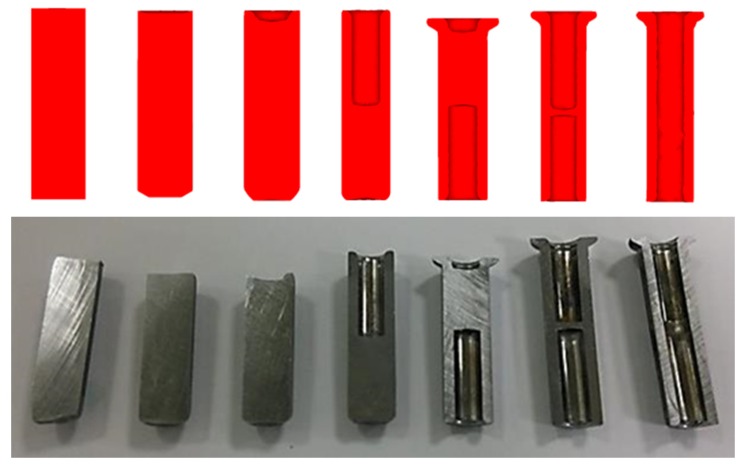
Workpieces of the simulation and actual long hex flange nuts from the original billet and the first to sixth stages.

The product was tested in a small batch production. Figure 19 compares the workpiece simulated at various stages and actual forge forming where their geometries are similar. Table 7 compares the DEFORM simulation with the actually produced sizes, with an error within 1%, except for the 1.87% error at S5 caused by its small size. This finding demonstrates that the accuracy of new product development following DEFORM simulation is good. The dimensional accuracies obtained from experimental and numerical analyses appear to be consistent and trustworthy.

**Table 7:** Comparison of dimensions between simulation and experimental products.

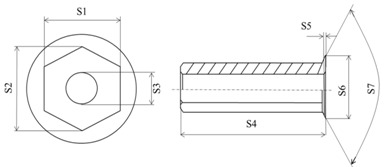			
	Exp.	FEM	Error (%)
S1 (mm)	11.96	11.90	−0.51
S2 (mm)	13.64	13.74	0.73
S3 (mm)	6.89	6.90	0.15
S4 (mm)	49.88	49.95	0.14
S5 (mm)	1.60	1.63	1.87
S6 (mm)	17.33	17.30	−0.17
S7 (°)	119.00	120.00	0.84

## 4. Conclusions

By using DEFORM-3D, this study conducted a fabrication analysis of a preform design of long hex flange nuts. Exactly how die wear, die stress, die interference and workpiece stress were related was also examined. Based on simulation and experimental results, we concluded the following:

(1) Introducing CAD/CAE to new fastener product forming and die design analysis can effectively shorten product development schedules and reduce the number of die testing intervals.
(2) Maximum wear depth and the position of stress concentration between die and workpiece can be determined through die action and workpiece flow during the third and fifth stages. In these cases, we recommend using tungsten steel for longer-wearing punches having reduced costs.
(3) Optimization analysis of the upper punch during the third stage indicated a potential for 19.87% improvement in die life. The arc length of the upper punch was the primary impact factor, followed by flat top, ring height, outer diameter and oblique angle.
(4) SEM micrographs of worn surfaces indicated that adhesive wear may be the primary wear mechanism.
(5) In comparing simulation *versus* real production sizes, the nut forging size error was 2%, thereby demonstrating the accuracy of the simulated forming process.

With the DEFORM-3D finite element analysis simulation and small-scale production testing, cold-forged automotive repair fasteners can be introduced into automatic production to reduce prohibitively expensive labor costs. This can also enhance production and quality and reduce material and equipment losses.
